# Factors associated with farrowing assistance in hyperprolific sows

**DOI:** 10.5713/ab.23.0169

**Published:** 2023-08-30

**Authors:** Napatsawan Wongwaipisitkul, Yanwarut Chanpanitkit, Natthacha Vaewburt, Piyakorn Phattarathianchai, Padet Tummaruk

**Affiliations:** 1 Department of Obstetrics, Gynaecology, and Reproduction, Faculty of Veterinary Science, Chulalongkorn University, Bangkok, 10330, Thailand; 2 Centre of Excellence in Swine Reproduction, Chulalongkorn University, Bangkok, 10330, Thailand

**Keywords:** Dystocia, Hyperprolific Sows, Hypoxia, Parturition, Reproduction

## Abstract

**Objective:**

The present study was performed to determine risk factors associated with the frequency of farrowing assistance in hyperprolific sows in a tropical environment and to investigate the impacts of farrowing assistance on piglet colostrum consumption and sow colostrum yield.

**Methods:**

Farrowing data from 352 Landrace×Yorkshire crossbred sows and 5,554 piglets in five commercial swine herds in Thailand were investigated. The sows were classified according to parity numbers: 1 (n = 72), 2 to 4 (n = 128), 5 to 6 (n = 84), and ≥7 (n = 68) and the total number of piglets born per litter (TB): 10 to 13 (n = 90), 14 to 16 (n = 117), and ≥17 (n = 145). The incidence of farrowing assistance and associated parameters were investigated.

**Results:**

The TB and farrowing duration averaged 15.8±0.2 and 279.9±11.2 min, respectively. The percentage of sows that required farrowing assistance was 29.8% and varied among herds from 5.7% to 53.3% (p<0.001). The percentage of piglets born after birth assistance using manual intervention was 8.4%. Sows with parity numbers 1 and 2 to 4 had a lower frequency of farrowing assistance than sows with parity numbers ≥7 (p<0.01). The colostrum yield of sows that required farrowing assistance did not differ from sows that farrowed without assistance (5.3±0.2 and 5.1±0.1 kg; p = 0.288); however, the colostrum consumption of piglets born from sows that required farrowing assistance was lower than those born from sows that farrowed without assistance (302.2±15.7 and 354.2±5.6 g; p<0.001). Blood oxygen saturation of the piglets born after birth assistance tended to be lower than the piglets that farrowed without birth assistance (87.8%±1.3% vs 90.4%±0.4%; p = 0.054).

**Conclusion:**

The frequency of farrowing assistance in sows varied among herds and was influenced by parity number. The piglets born after receiving birth assistance should receive special care to improve their blood oxygen saturation and enhance colostrum intake.

## INTRODUCTION

In the past decades, the genetic potential on litter size at birth of breeding sows used in most swine commercial herds worldwide has been successfully improved. Thus, the total number of piglets born per litter (TB) of sows in the modern swine industry has dramatically increased over the last decade [1–3]. However, large litter size also causes some problems, including prolonged farrowing duration, low birth weight piglets, inadequate colostrum consumption, high stillbirth rate, and an increased incidence of birth assistance [4,5]. These problems led to an increase in the research interest to investigate effective management strategies to reduce clinical problems in these modern hyperprolific sows [6–8]. A previous study from the USA indicated that the incidence of farrowing assistance was as high as 17.2% in sows with an average TB of 16.9 piglets per litter [9]. In Denmark, clinical research has found that 24% of sows that had average TB of 17.5 require farrowing assistance at least once during parturition [10]. Moreover, the incidence of farrowing assistance was positively correlated with farrowing duration and the percentage of stillbirths [10]. The farrowing duration is prolonged by 0.72 h when farrowing assistance is performed [10]. When the time elapsed from the last meal until the onset of farrowing increased from ≤3 to >6 h, the demand for farrowing assistance rose ninefold [10]. In addition, keeping sows in farrowing crates also causes stress and, consequently, elevated plasma cortisol levels, which negatively influences oxytocin function and compromise uterine contraction [11]. Cortisol concentrations in sows are positively correlated with farrowing duration [12]. Furthermore, Björkman et al [13] found that the farrowing duration is significantly increased in old sows. Similarly, in tropical areas, Adi et al [14] have demonstrated that the farrowing duration of sows with parity numbers 5 to 7 and 8 to 10 was longer than that of sows with parity numbers 1 and 2 to 4. Therefore, stress, as well as aging (i.e., high parity number), possibly affected the farrowing duration of sows.

Dystocia is defined as a slow or difficult parturition due to fetal obstruction in the birth canal or inadequate myometrial contractions, which subsequently require farrowing assistance [15]. In modern hyperprolific sows, dystocia is becoming an interesting issue in swine research due to a long duration of farrowing and high incidence of stillbirths [1,16]. However, in practice, performing farrowing assistance in peri-partum sows may interfere with their natural process of parturition and can be one of the stressful factors causing postpartum complications. Theoretically, cortisol secretion causes a decrease in oxytocin secretion and may compromise the colostrum and/or milk production of sows [11]. A previous study has demonstrated that an additional minute of farrowing duration can reduce the colostrum yield by 2.2 g [17]. However, comprehensive study concerning the direct effect of farrowing assistance on the colostrum production of sows has not been demonstrated. Moreover, dystocia can lead to poor piglet outcome and health problems in sows associated with both economic and animal welfare issues in the modern swine industry [15]. Although the incidence of dystocia, as well as its consequence on both sows and piglet performance, is an important issue in modern hyperprolific sows, research in this area is still lacking [15]. Therefore, the present study was carried out to determine risk factors associated with the incidence of farrowing assistance (i.e., a consequence of dystocia) in hyperprolific sows in commercial swine herds in tropical environments. Additionally, the impact of farrowing assistance on piglet colostrum consumption and sow colostrum yield was also evaluated.

## MATERIALS AND METHODS

### Experimental design

The results presented in this paper are based on data generated from five independent studies on five commercial swine herds (namely herds A, B, C, D, and E) in Thailand during 2014 through 2020 [7,18–21]. The previous studies were conducted to assess the effects of various hormonal treatments, including prostaglandin F2alpha (PGF2α) [20], oxytocin [19] and carbetocin [7], on farrowing performance, characteristics of newborn piglets and colostrum intake. However, the potential impact of farrowing assistance on farrowing performance and piglet characteristics has not been investigated. In order to mitigate the potential confounding effects of these hormones on the incidence of farrowing assistance and farrowing kinetics, the current study excluded sows that had received PGF2α [20], oxytocin [19], or carbetocin [7] before or during the parturition process. Furthermore, in other previous studies [18,21], the objective was to investigate the impact of feed additives to enhance sows’ appetite during the postpartum period, and their farrowing characteristics were not analyzed. All experiments were carried out in accordance with the ethical principles and guidelines for the use of animals in scientific research, as outlined by the National Research Council of Thailand. The research proposals were approved by the institutional animal care and use committee, adhering to Chulalongkorn University’s regulations and policies governing the care and use of experimental animals [7,18–21]. In the present study, the data included 352 sows and their offspring (n = 5,554) farrowed naturally without any parturition induction. The sows were classified into four groups according to their parity number: 1 (n = 72), 2 to 4 (n = 128), 5 to 6 (n = 84), and ≥7 (n = 68). The litters were also classified into three groups according to the TB: 10 to 13 (n = 90), 14 to 16 (n = 117), and ≥17 (n = 145). The incidence of farrowing assistance, farrowing duration, birth interval, and percentage of stillborn piglets per litter were investigated. In addition, the reproductive performance of sows, colostrum yield, colostrum intake, and piglet characteristics were compared between sows or piglets that required birth assistance and those that were born naturally without assistance.

### Animals, housing, general management, and farrowing supervision

All the experiments were conducted following the guidelines documented in the ethical principles and guidelines for the use of animals for scientific purposes edited by the national research council of Thailand and were approved by the institutional animal care and use committee in accordance with Chulalongkorn University regulations and policies governing the care and use of experimental animals. In all herds, the sows were kept in individual stalls (1.2 m^2^) during gestation and were moved to a farrowing room and allocated to individual farrowing crates (1.5 m^2^) placed at the center of pens with a space allowance of 4.2 m^2^ at 109±2 days of gestation. In herds A, D, and E, the sows were kept in a closed-housing system equipped with an evaporative cooling system and temperature control facilities to maintain the optimal temperature inside the barn. The ambient temperature inside the barn during the experimental period was 29.0°C±1.78°C (ranged from 24.2°C to 31.2°C) and the relative humidity inside the barn varied from 74.0% to 89.0%. In herds B and C, the sows were kept in a conventional open-housing system and were provided with fans and individual water sprinklers to reduce the impact of high ambient temperature. The ambient temperature during the experimental period ranged from 25.8°C to 30.0°C and the relative humidity varied from 72.0% to 96.0%. During gestation, sows were fed a gestation diet twice a day following a standardized feeding pattern, resulting in 1.8 to 4.5 kg of feed per sow per day to meet or exceed their nutritional requirements [22]. The feed offered to sows was reduced to 2.0 kg for one day before farrowing. After farrowing, the sows were fed a standard lactation diet [22] and the amount of feed offered to the sows increased by 0.5 kg per day until *ad libitum* feed was reached after one week of lactation. Sows and piglets had *ad libitum* access to water via nipple drinkers. The parturition process was continuously supervised by the researcher teams for 24 h daily. During parturition, the sows were interfered with as little as possible. Manual intervention during the birth process was done only when dystocia was clearly observed. Dystocia was considered when an interval of 30 to 60 min elapsed from the birth of the last piglet, or when the sow showed intermittent straining accompanied by paddling of the legs or when the sow expelled small quantities of fetal fluid together with marked tail switching for 30 to 60 min without any piglet being born [19]. The birth assistance included the stimulation of uterine contraction by palpating dorsal wall of vagina (Ferguson reflex), manual extraction of the piglets and, in some cases, intramuscular administration of 10 IU oxytocin.

### Data

The number of sows in herds A, B, C, D, and E were 45, 31, 73, 105, and 98, respectively. The parity number of sows averaged 4.1±0.1 and ranged from 1 to 12. Sow data, including the sow identity, parity number, backfat thickness at 109 days of gestation, TB, number of piglets born alive per litter (BA), number of mummified fetuses per litter (MF), number of stillborn piglets per litter (SB), and farrowing duration, were collected. Farrowing assistance was considered a binomial trait, defined as ‘0’ when the sows could farrow completely without any birth assistance by manual extraction, and defined as ‘1’ when the sows required farrowing assistance through manual intervention during the parturition process. The incidence of farrowing assistance was expressed as a percentage. Farrowing duration was defined as the time interval from the first to the last piglet born in minutes. The inter-piglet interval or birth interval was defined as the time between the expulsion of one piglet and the next in minutes. Backfat thickness was measured at the P2 position on the right and left side of the sow (6 cm from the midline, straight above the last rib bone [23] on day 109±2.0 of gestation using A-mode ultrasonography (Renco Lean-Meater, Minneapolis, MN, USA).

The number of piglets in herds A, B, C, D, and E was 568, 443, 1,077, 1,832, and 1,634, respectively. The piglet data included body weight at birth and at 24 h after birth, birth interval, cumulative birth interval (i.e., the accumulation time from the onset of second stage of parturition until delivery), blood oxygen saturation (SatO2), and their colostrum intake. Blood oxygen saturation was assessed within 5 min of birth using a pulse oximeter (Edan Instruments Inc., Nanshan Shenzhen, China). The sensor ear clip of the pulse oximeter was positioned on the middle part of the piglets’ ears after clearing away the amniotic fluid [7]. The body weight of the piglet was determined twice, at birth (i.e., before colostrum ingestion) and 17 to 24 h after birth, using a digital balance (SDS IDS701-CSERIES; SDS Digital Scale Co., Ltd., Yangzhou, China). The colostrum intake of each piglet was estimated with the previously described equation by Theil et al [24]: Colostrum intake (g) = −106+2.26WG+200BW_B_ +0.111D–1,414WG/D+0.0182WG/BW_B_, where WG is piglet weight gain over 24 h (g), BW_B_ is birth weight (kg), and D is the duration of colostrum suckling (min). Colostrum yield of sows was determined as the summation of the colostrum intake of all piglets in the litter. The colostrum yield, colostrum intake, and all piglet characteristics were calculated and compared between sows or piglets that required birth assistance and those that were born naturally without assistance.

### Statistical analyses

The statistical analyses were performed using SAS version 9.4 (SAS Inst Inc, Cary, NC, USA). Descriptive statistics, including mean, standard error of the mean (SEM), and range of the data, were carried out to analyse continuous data. For sows (n = 352), continuous variables, including backfat thickness at 109 days of gestation, farrowing duration, TB, BA, SB, MF, colostrum yield, litter birth weight, variation of piglet birth weight within the litter, and variation of colostrum consumption of piglets within the litter, were analysed by multiple analysis of variance using the general linear model (GLM) procedure of SAS. The statistical models included the effect of herds (A, B, C, D, and E), parity number classes (1, 2 to 4, 5 to 6, and ≥7), TB classes (10 to 13, 14 to 16, and ≥17), farrowing assistance (yes, no), and two-way interactions. The factors and interactions included in the statistical models were tested for significance and then stepwise omitted from the model. For piglets (n = 5,554), the continuous variables included birth interval (min), cumulative birth interval (min), SatO2 (%), piglet birth weight (kg), piglet body weight at 24 h after birth (kg), and colostrum consumption (g) were compared between piglets born with and without assistance using the general linear mixed model (MIXED) procedure of SAS. The statistical models included the fixed effect of herds (A, B, C, D, and E), parity number classes (1, 2 to 4, 5 to 6, and ≥7), TB classes (10 to 13, 14 to 16, and ≥17), birth assistance (yes, no), and two-way interactions. The sow identity was also included in the statistical model as a random effect to adjust for the repeated measurement. Least-square means were obtained from the statistical models and were compared using the least significant difference (LSD) test.

Frequency analysis was conducted to analyze categorical data using PROC FREQ of SAS. Logistic regressions were conducted to analyze the binomial traits, including farrowing assistance in sows (n = 352) and birth assistance in piglets (n = 5,554), using the generalized linear mixed model (GLIMMIX) procedure of SAS. For sows, the statistical model included the effect of herds (A, B, C, D, and E), parity number classes (1, 2 to 4, 5 to 6, and ≥7), TB classes (10 to 13, 14 to 16, and ≥17), and two-way interactions. For piglets, the statistical model included the effect of herds (A, B, C, D, and E), parity number classes (1, 2 to 4, 5 to 6, and ≥7), TB classes (10 to 13, 14 to 16, and ≥17), and two-way interactions. The sow identity was also included in the statistical model as a random effect. Least-square means were obtained from each class of the factors and were compared using the LSD test. The arithmetic means±SEM for all variables included in the current study are displayed in Table 1. However, the information presented in Tables 2 to 5 utilizes least-square means ±SEM, as opposed to arithmetic mean or general mean. This choice stems from the capability of least-square means to accommodate unevenly distributed observations among the different groups and sub-groups, enabling a more equitable comparison. Consequently, these least-square means were generated from the complete set of statistical models employed in this study. The differences with p<0.05 were regarded as statistically significance.

## RESULTS

### Descriptive statistics

**Figure 1:**
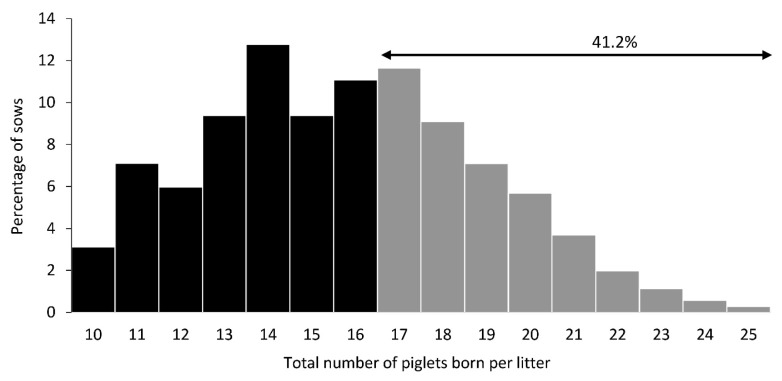
Frequency distribution of the total number of piglets born per litter in commercial swine herds in Thailand (n = 352 sows).

Descriptive statistics on reproductive performance of sows and piglet characteristics are presented in Table 1. Across herds, the average TB, BA, SB, and MF were 15.8±0.2, 13.8± 0.2, 1.2±0.1, and 0.7±0.1, respectively. Frequency distribution of TB is presented in Figure 1 and 41.2% of the sow had ≥17 TB. On average, the farrowing duration of sows across herds was 279.9±11.2 min, but it varied among individual sows, ranging from 53 to 1436 min (Table 1). The mean piglet birth weight was 1.29±0.005 kg, and the mean colostrum intake was 344.6±2.8 g. Colostrum production of sows averaged 5.3±0.1 kg.

### Frequency of farrowing assistance

The reproductive performance of the sows and piglet characteristics of each herd are presented in Table 2. The percentage of sows that required farrowing assistance at least once was 29.8% (105/352 sows) and varied among herds from 5.7% to 53.3% (p<0.001; Table 2). Among the sows that required farrowing assistance (n = 105), the number of piglets requiring birth assistance per litter averaged 4.4±0.3 and ranged from 1 to 16. Of the litters that required farrowing assistance (n = 105), the proportion of sows that required birth assistance for 1, 2, 3, and ≥4 piglets were 21.0%, 19.1%, 16.2%, and 43.7%, respectively. For piglets, the percentage of piglets that required birth assistance was 8.4% (465/5,554 piglets) and varied from 0.7% to 15.9% (p<0.001; Table 2).

The frequency of farrowing assistance differed significantly among sow parity groups, ranging from 22.2% to 52.9% (Table 3). Sows with parity numbers 1 and 2 to 4 had a lower frequency of farrowing assistance than sows with parity numbers ≥7 (p<0.01), but did not differ significantly compared to sows with parity numbers 5 to 6 (Table 3). Similarly, among the sows that required farrowing assistance, the number of piglets that required birth assistance within the litters in sows with parity numbers ≥7 was also higher than sows with parity numbers 5 to 6 (Table 3). The frequency of farrowing assistance and reproductive performance of sows by TB classes are presented in Table 4. The frequency of farrowing assistance did not differ significantly between the litters with 10 to 13, 14 to 16, and ≥17 TB (Table 4).

### Farrowing duration and birth interval

The mean farrowing duration showed variation among herds, ranging from 201.9 to 346.4 min (Table 2). On average, the farrowing duration in sows with parity numbers ≥7 was longer than primiparous sows and sows with parity numbers 2 to 4 and 5 to 6 (Table 3). Likewise, the birth interval, as well as cumulative birth interval of piglets in primiparous sows, was shorter than sows with parity numbers 2 to 4, 5 to 6, and ≥7 (p<0.05; Table 3). Farrowing duration in the litters with ≥17 TB was longer than the litters with 14 to 16 TB but did not differ significantly compared to the litter with 10 to 13 TB (Table 4). Similarly, the cumulative birth interval of piglets in the litters that had ≥17 TB was longer than the litters with 10 to 13 and 14 to 16 piglets born per litter (Table 4). On the other hand, the birth interval of piglets in the litters that had ≥17 TB was shorter than the litters with 10 to 13 piglets born per litter (Table 4).

### Colostrum yield and intake

The average colostrum yield varied from 4.8 to 5.5 kg (p< 0.05) among sow parity groups, with the lowest in primiparous sows and the highest in sows with parity numbers 2 to 4 (Table 3). Primiparous sows had a lower colostrum yield than sows with parity numbers 2 to 4 and 5 to 6 but did not differ significantly from sows with parity numbers ≥7 (Table 3). Likewise, colostrum consumption of piglets in primiparous sows was lower than piglets in sows with parity numbers 2 to 4 and 5 to 6 but did not differ significantly compared to piglets from sows with parity numbers ≥7 (Table 3). The average colostrum yield of sows that had 10 to 13 TB was lower than sows that had ≥17 TB but did not differ significantly compared to the litter that had 14 to 16 TB (Table 4). On the other hand, the average colostrum consumption of piglets in the litters that had 10 to 13 and 14 to 16 TB was significantly higher than that of the piglets in the litters that had ≥17 TB (Table 4).

### Characteristics of sows and piglets that required farrowing assistance

The characteristics of sows and piglets that had normal parturition compared to those that required farrowing assistance are presented in Table 5. Litter traits, including TB, BA, SB, and MF, in the sows that required farrowing assistance did not differ significantly compared to the sows that farrowed without assistance (p>0.05); however, the farrowing duration of sows that required assistance was longer than sows that farrowed without assistance (308.7±23.9 vs 241.5±15.7 min, respectively; p = 0.019). The colostrum yield of sows that required farrowing assistance did not differ significantly compared to sows that farrowed without assistance (5.3±0.2 vs 5.1±0.1 kg, respectively; p = 0.288).

Piglets that required farrowing assistance had a longer birth interval than piglets that did not require farrowing assistance (20.6±1.8 vs 13.8±0.9 min, respectively; p<0.001). Similarly, the cumulative birth interval of piglets that required farrowing assistance was longer than piglets that did not require assistance (169.0±8.7 vs 109.4±4.2 min, respectively; p<0.001). The colostrum consumption of piglets born from sows that required farrowing assistance was lower than that from sows who farrowed without assistance (302.2±15.7 vs 354.2±5.6 g, respectively; p<0.001). Interestingly, blood oxygen saturation in the piglets that required farrowing assistance tended to be lower than in piglets that farrowed without assistance (87.8%±1.3% vs 90.4%±0.4%; p = 0.054).

## DISCUSSION

Farrowing problems (dystocia) not only impact the health of sows and their piglets but also result in economic losses for swine herds. Additionally, this issue has emerged as a significant welfare concern within the pig industry [15]. Despite its significance, there is still a dearth of publications in this area over the past 50 years [15]. The present study aims to address the importance of farrowing duration and the incidence of farrowing assistance in hyperprolific sows within tropical environments. It is crucial to acknowledge that the sows in this study experienced moderate to severe heat stress, as the temperature inside the farrowing house across all herds ranged from 24.2°C to 31.2°C, surpassing the upper limit of the sows’ thermoneutral zone [3,25]. On average, the farrowing duration of sows in this study was 280 min, with variations among herds ranging from 202 to 346 min.

### Farrowing assistance

The present study demonstrated the frequency of farrowing assistance in five commercial swine herds in Thailand. Across the herds, 29.8% of the sows required farrowing assistance; however, a huge variation in the frequency of farrowing assistance was observed among herds (from 5.7% to 53.3%). The variation among herds could be attributed to single or multiple factors, such as the herd policy for farrowing intervention, varied decisions made by stockpersons, the body condition score of sows, the proportion of old sows, constipation status, feed and feeding practices during transition periods, the level of fiber in the feed, and housing system [7,10,14]. For example, in the current study, herd D exhibited a relatively low incidence of farrowing assistance. This is attributed to the herd’s policy of minimizing manual piglet extraction during parturition, with all farrowing interventions strictly overseen by a veterinarian. In contrast, other herds may rely on experienced animal husbandry personnel to handle such procedures. The high prevalence of farrowing assistance in the majority of herds observed in this study aligns with findings from a recent study conducted in Vietnam [26]. The study indicated that dystocia was prevalent in Landrace × Yorkshire crossbred sows in commercial swine herds, with a rate as high as 47.0% under tropical environmental conditions [26]. In Denmark, a clinical study based on 166 litters found that 24% of the sows received assistance at least once during farrowing [10]. The lower frequency of farrowing assistance in the Danish swine herd, as compared to the swine herds in the present study, might be due to the fact that the general recommendation is that farrowing assistance will be performed when the interval between two consecutive births exceeds 90 min [10]. In Thailand, birth assistance, including the stimulation of uterine contraction by palpating the dorsal wall of the vagina (Ferguson reflex), manual extraction of the piglets, and, in some cases, administration of 10 to 20 IU oxytocin, are usually performed when an interval of 30 to 60 min has elapsed since the birth of the last piglet [19]. The average birth interval of piglets in the Danish herd was 21.6 min [10], similar to the birth interval observed in the present study (18.9 min); however, we demonstrated that the average birth interval of sows that required birth assistance was longer than that of the sows that farrowed without assistance (20.6 vs 13.8 min, respectively). The increase of farrowing duration as well as birth interval may be associated with repeated uterine contractions and compression of the placental blood supply, resulting in reduced fetal oxygenation [27]. This condition can even be worsened when the farmers are trying to speed up the prolonged farrowing by administrating the sow with repeated or too-high dosages of oxytocin [28]. In the present study, most of the sows required farrowing assistance also received oxytocin administration during farrowing, some of them up to four times. This can increase the risk of dystocia due to the saturation of uterine oxytocin receptors [29]. Moreover, we also demonstrated that the number of piglets per litter that required birth assistance averaged 4.2 and varied from 1 to 16. Among the sows that required birth assistance, most of them (i.e., 42.2%) needed manual intervention for ≥4 piglets. Interestingly, farrowing assistance was more frequently observed in sows with parity numbers ≥7 than in young sows. This indicates a negative impact of aging on the farrowing performance of sows. This finding agrees with Adi et al [14], who demonstrated that the average farrowing duration in old sows was nearly one hour longer than in young sows. These findings indicated that additional knowledge on the risk factors associated with poor uterine contraction, as well as a practical solution to solve this problem in old sows, should be focused on.

### Colostrum

In the present study, the colostrum consumption of piglets born after birth assistance was significantly lower than the piglets farrowed without assistance. This could be explained by the results observed in the present study, which indicated that blood oxygen saturation in the piglets born after birth assistance was lower than those born without farrowing assistance. Moreover, the birth interval, as well as the cumulative birth interval, in the piglets born after birth assistance were significantly longer than that of the piglets born without assistance. This indicates that piglets that had birth assistance might have suffered from a long duration of uterine contractions, which could have led to hypoxia. The piglets that had hypoxia had a lower ability to ingest colostrum than normal piglets [30]. Supplementation of oxygen immediately after birth in newborn piglets using a specially designed facemask for 45 s to 1 min significantly improved blood oxygen saturation from 86.8% to 97.8% and increased colostrum consumption of piglets from 273.2 to 401.6 g [30]. This finding indicates that oxygen supplementation can be one practical solution to improve colostrum consumption of the piglets born after manual intervention. Moreover, we also observed that the birth weight of piglets born after birth assistance was higher than the piglets born without assistance. Although the birth weight of the piglets is positively associated with colostrum consumption [5], high birth weight piglets that experience dystocia and required birth assistance is an exceptional case. Thus, special care of high birth weight piglets that required birth assistance may help to improve their colostrum consumption.

### Inter-piglet interval

Normally, the sows farrow with the inter-piglet interval of 14 to 20 min [1,19]. In the present study, the birth interval of piglets that required birth assistance was longer than piglets born without assistance. The long birthing interval could be due to sow exhaustion and reduced uterine contraction intensity. Piglets who experienced a long birth interval may have a high risk of mortality due to hypoxia. Udomchanya et al [1] have demonstrated that the birth interval of stillborn piglets was longer than live-born piglets (33.0 vs 13.9 min, respectively). In practice, manual extraction of the foetuses is generally advised if the birth interval exceeds 45 min [16]; however, a gold standard for manual intervention of piglets is still controversial [10,15]. Interestingly, in the present study, the number of stillborn piglets did not differ significantly between piglets born with and without birth assistance. In a previous study, a reduction in stillbirths was observed when manual intervention was undertaken [31]. This finding indicates that manual intervention may be needed to avoid the high incidence of stillbirths and live-born piglets that had hypoxia in hyperprolific sows; however, the consequences of manual intervention, often by nonsterile techniques, are still questionable [15].

### Sow parity numbers

The present study found that 19.8% of the piglets born from sows with parity numbers ≥7 required birth assistance and 52.9% of these sows required farrowing assistance at least once. Moreover, sows with parity numbers ≥7 had the longest farrowing duration (i.e., 346.4 min) and the highest number of stillborn piglets (i.e., 1.8 piglets per litter). Adi et al [14] have demonstrated that the farrowing duration of sows with parity numbers 5 to 7 and 8 to 10 was longer than that of sows with parity numbers 1 and 2 to 4, and the farrowing duration was positively correlated with the percentage of stillborn piglets per litter. The percentage of stillborn piglets per litter in sows with parity numbers 8 to 10 (8.9%) was higher than that of primiparous sows (3.8%) and sows with a parity number of 2 to 4 (4.4%) [14]. Moreover, longer farrowing duration can be detrimental and negatively influence the colostrum yield of sows [17]. One explanation for the relationship between sow parity number and farrowing duration could be that prolonged farrowing duration (>240 min) was associated with a lower circulating oxytocin concentration [12]. Moreover, Olmos-Hernández et al [32] have shown that primiparous sows exhibited higher frequencies and intensities of myometrial contractions compared to sows with parity numbers 3 to 6. In mice, the myometrium of old mice showed a reduction in the oxytocin receptor levels compared to young mice [33]. Therefore, old sows that had a long farrowing duration might have a high frequency of farrowing assistance and have a poor colostrum yield due to low circulating oxytocin. Moreover, the long duration of farrowing in sows with high parity numbers might also be associated with the aging of the uterine muscle tissue after multiple births and the lower intensity uterine contractions [34]. This could also compromise the farrowing process and increase the risk of dystocia in sows with high parity numbers. Therefore, intensive farrowing supervision, as well as the proper use of oxytocin, in sows with parity numbers ≥7 should be raised to reduce the incidence of stillbirths and enhance the colostrum consumption of piglets. Indeed, the proper use of oxytocin in parturient sows to reduce the duration of labor has been published [19,35]. The mortality rate of piglets born to sows exposed to oxytocin during parturition was modified by the time at which the hormone was administered during parturition; the mortality rate was higher when oxytocin was administered after the birth of the 1st piglet compared to that administered after the 4th or the 8th piglet [35].

### Litter size

Surprisingly, the frequency of farrowing assistance did not differ significantly among different classes of litter size. The frequency of farrowing assistance was 21.4% in sows with ≥17 TB and 35.6% to 35.9% in sows with 10 to 16 TB; however, we found that litter size influenced the farrowing duration of sows. Sows that had TB ≥17 had an average farrowing duration of 5.2 h, while sows that had 10 to 16 TB had a farrowing duration of 4.0 to 4.6 h. Interestingly, the inter-piglet interval was shorter in the litters with ≥17 TB than the litters with 10 to 13 TB (i.e., 16.2 vs 20.0 min, respectively). This could be because the average birth weight of the piglets in the litters with ≥17 TB was lower than the litters with 10 to 13 TB (i.e., 1.21 vs 1.41 kg, respectively). Large piglets take a longer time to be expelled compared to the smaller ones [15,31]. Moreover, although having a better vitality, large piglets have increased difficulties engaging in the vaginal canal and a greater risk of being obstructed in the birth canal, which can lead to hypoxia and increase the risk of dying or requiring farrowing assistance. Therefore, the longer inter-piglet interval in the small litters (i.e., 10 to 13 TB) could be explained by the larger size of the piglets. Therefore, the reason why litter size had no significant impact on the frequency of farrowing assistance, though the farrowing duration was longer, could be due to the smaller size of piglets which lead to easier expulsion. Nevertheless, the prolonged farrowing duration in the litters with ≥17 piglets is concerning because it can be a risk of severe postpartum complications in sows. In Denmark, where the TB is 19, the average farrowing duration has been reported to be as high as 9.7 h [36]. We also found that number of stillbirths in the sows that had >17 TB was higher than the sows that had 10 to 16 TB (i.e., 2.0 vs 0.7 to 0.8 SB), which agrees with earlier studies [1,37]. These data indicated that, although litter size had no negative impact on dystocia, the prolonged uterine contraction due to large litter size can compromise vitality of fetuses by reducing blood flow to the placenta, leading to hypoxia and death. Moreover, low birth weight of the newborn piglets due to large litter size can also compromise their colostrum consumption [5]. In the present study, piglets born in the litters with 10 to 13 TB had a 32.0% higher colostrum consumption than the piglets born in the litter with ≥17 TB (i.e., 385.0 vs 268.9 g, respectively). Therefore, although large litter size had no negative impact on the frequency of farrowing assistance, it affects farrowing duration, number of stillbirths, and colostrum consumption of the newborn piglets.

## CONCLUSION

In conclusion, 29.8% of hyperprolific sows in commercial swine herds under tropical environments required farrowing assistance, and 8.4% of the piglets were born after manual intervention. The frequency of farrowing assistance in sows varied among herds from 5.7% to 53.3% and was influenced by parity number but not litter size. Across herds, 52.9% of sows with parity numbers ≥7 required birth assistance, and 19.8% of their offspring were born after manual intervention. Piglets born with birth assistance exhibited lower blood oxygen saturation and colostrum intake compared to piglets born without assistance. As a result, these piglets require special attention to enhance their blood oxygen saturation and increase colostrum intake. Additionally, in order to decrease the need for farrowing assistance, it is advisable to minimize the number of sows with parity numbers ≥7.

## Figures and Tables

**Table 1:** Descriptive statistics on sow reproductive performance and piglet characteristics

Variables	Means±SEM	Range
Sows (n = 352)		
Parity number	4.1±0.1	1–12
Backfat thickness at 109 days of gestation (mm)	15.0±0.2	6–32
Farrowing assistance (%)	29.8	-
Farrowing duration (min)	279.9±11.2	53–1436
Total number of piglets born per litter	15.8±0.2	10–25
Number of piglets born alive per litter	13.8±0.2	0–22
Number of stillborn piglets per litter	1.2±0.1	0–10
Number of mummified fetuses per litter	0.7±0.1	0–15
Colostrum yield (kg)	5.3±0.1	0–9.1
Piglets (n = 5,554)		
Birth interval (min)	18.9±1.0	0–1086
Cumulative birth interval (min)	136.8±2.0	0–1820
Birth weight (kg)	1.29±0.005	0.20–2.85
Body weight at 24 h after birth (kg)	1.40±0.005	0.29–2.83
Colostrum consumption (g)	344.6±2.8	0–998
Variation of piglet birth weight (% CV)	21.7±0.3	1.8–40.7
Variation of colostrum consumption (% CV)	53.1±1.0	8.4–97.4

SEM, standard error of the mean; CV, coefficient of variation.

**Table 2:** Incidence of farrowing assistance and reproductive performance of sows in five commercial swine herds in Thailand (least square means±SEM)

Variables	Herd				

	A	B	C	D	E
Number of sows	45	31	73	105	98
Parity number	5.1±0.1^a^	4.1±0.2^b^	4.2±0.1^b^	4.2±0.2^b^	4.4±0.1^b^
Backfat thickness at 109 days of gestation (mm)	16.5±0.5^a^	16.0±0.7^ab^	14.6±0.4^b^	14.1±0.8^c^	NA
Farrowing duration (min)	201.9±32.2^a^	227.8±36.7^a^	253.4±22.9^ab^	346.4±46.1^b^	346.0±22.6^b^
Farrowing assistance (%)	53.3^a^	41.9^a^	42.5^a^	5.7^c^	31.6^b^
Number of birth assistance	3.4±0.9^a^	3.4±1.0^a^	5.3±0.6^a^	2.1±1.6^a^	4.5±0.7^a^
TB	14.9±0.2^ab^	14.9±0.3^ab^	15.0±0.2^a^	15.7±0.3^b^	15.3±0.2^ab^
BA	13.6±0.4^a^	12.1±0.4^b^	12.9±0.3^ab^	13.9±0.5^a^	13.2±0.3^a^
SB	1.0±0.2^ab^	1.6±0.3^a^	1.3±0.2^a^	0.5±0.3^b^	1.5±0.2^a^
MF	0.3±0.2^a^	0.9±0.2^ab^	0.6±0.2^a^	1.3±0.3^b^	0.5±0.1^a^
Colostrum yield (kg)	5.0±0.2^a^	4.6±0.3^a^	5.2±0.2^a^	6.0±0.3^b^	5.1±0.2^a^
Number of piglets	568	443	1,077	1,832	1,634
Birth interval (min)	18.4±1.5^ab^	22.1±2.0^a^	17.8±1.0^ab^	10.8±3.8^b^	17.0±1.1^b^
Cumulative birth interval (min)	157.2±11.9^a^	120.3±14.5^ab^	145.4±8.7^ab^	149.6±17.5^ab^	123.5±8.4^b^
Birth assistance (%)	15.9^a^	9.1^b^	15.5^a^	0.7^c^	9.6^b^
Birth weight (kg)	1.23±0.03^a^	1.33±0.04^b^	1.44±0.02^c^	1.20±0.05^a^	1.29±0.02^a^
Body weight at 24 h (kg)	1.35±0.03^a^	1.43±0.04^ab^	1.52±0.02^b^	1.33±0.06^a^	1.38±0.02^a^
Blood oxygen saturation (%)	92.4±1.0^a^	90.2±1.2^ab^	87.6±0.8^b^	86.2±2.2^b^	NA
Colostrum consumption (g)	319.2±17.0^a^	321.8±22.2^a^	348.5±12.4^a^	322.2±30.6^a^	329.3±12.4^a^

SEM, standard error of the mean; NA, data not available; TB, total number of piglets born per litter; BA, number of piglets born alive per litter; SB, number of stillborn piglets per litter; MF, number of mummified fetuses per litter.

a–c Different superscripts within each row differ significantly (p<0.05).

**Table 3:** Incidence of farrowing assistance and reproductive performance of sows by parity number (least square means±SEM)

Variables	Parity number			

	1	2 to 4	5 to 6	≥7
Number of sows	72	128	84	68
Backfat thickness at 109 days of gestation (mm)	14.1±0.5^a^	15.5±0.4^b^	16.5±0.5^b^	15.2±0.6^ab^
Farrowing duration (min)	228.5±26.0^a^	254.7±20.4^a^	270.8±25.1^a^	346.4±27.2^b^
Farrowing assistance (%)	22.2^a^	22.7^a^	28.6^ab^	52.9^b^
Number of birth assistance	3.1±0.9^ab^	3.6±0.7^ab^	3.2±0.8^a^	5.1±0.7^b^
TB	15.0±0.2^ab^	15.2±0.1^ab^	15.5±0.2^a^	14.9±0.2^b^
BA	13.4±0.3^a^	13.7±0.2^a^	13.2±0.3^a^	12.2±0.3^b^
SB	0.9±0.2^ab^	0.7±0.2^a^	1.3±0.2^bc^	1.8±0.2^c^
MF	0.7±0.2^a^	0.6±0.1^a^	0.7±0.2^a^	0.8±0.2^a^
Colostrum yield (kg)	4.8±0.2^a^	5.5±0.1^b^	5.3±0.2^bc^	5.1±0.2^ac^
Number of piglets	988	2,093	1,437	1,024
Birth interval (min)	14.9±1.2^a^	18.2±1.1^b^	17.7±1.2^b^	18.2±1.2^b^
Cumulative birth interval (min)	118.2±9.4^a^	146.7±7.5^b^	147.9±9.0^b^	143.9±9.7^b^
Birth assistance (%)	5.2^a^	5.7^a^	6.4^b^	19.8^c^
Birth weight (kg)	1.18±0.02^a^	1.35±0.02^b^	1.33±0.02^b^	1.32±0.02^b^
Body weight at 24 h after birth (kg)	1.26±0.03^a^	1.46±0.02^b^	1.44±0.03^b^	1.45±0.03^b^
Blood oxygen saturation (%)	89.3±0.9^ab^	89.3±0.8^ab^	90.0±1.0^a^	87.7±1.0^b^
Colostrum consumption (g)	300.6±13.3^a^	347.0±11.0^b^	335.5±12.7^b^	329.7±13.5^ab^

SEM, standard error of the mean; TB, total number of piglets born per litter; BA, number of piglets born alive per litter; SB, number of stillborn piglets per litter; MF, number of mummified fetuses per litter.

a–c Different superscripts within each row differ significantly (p<0.05).

**Table 4:** Incidence of farrowing assistance and reproductive performance of sows by total number of piglets born per litter class (least square means±SEM)

Variables	Total number of piglets born per litter classes		

	10 to 13	14 to 16	≥17
Number of sows	90	117	145
Backfat thickness at 109 days of gestation (mm)	15.2±0.5^ab^	16.2±0.4^a^	14.6±0.5^b^
Farrowing duration (min)	275.8±23.5^ab^	238.8±20.8^a^	310.8±21.4^b^
Farrowing assistance (%)	35.6^a^	35.9^a^	21.4^a^
Number of birth assistance	3.2±0.8^a^	4.0±0.6^a^	4.1±0.7^a^
TB	12.0±0.2^a^	14.9±0.1^b^	18.6±0.1^c^
BA	10.4±0.3^a^	13.2±0.2^b^	15.7±0.3^c^
SB	0.7±0.2^a^	0.8±0.2^a^	2.0±0.2^b^
MF	0.7±0.2^a^	0.7±0.1^a^	0.8±0.1^a^
Colostrum yield (kg)	4.9±0.2^a^	5.3±0.1^ab^	5.4±0.1^b^
Number of piglets	1,059	1,744	2,739
Birth interval (min)	20.0±1.2^a^	15.4±1.1^b^	16.2±1.1^b^
Cumulative birth interval (min)	123.5±8.6^a^	127.9±7.5^a^	166.2±8.0^b^
Birth assistance (%)	11.1^a^	11.5^a^	5.4^a^
Birth weight (kg)	1.41±0.02^a^	1.27±0.02^b^	1.21±0.02^c^
Body weight at 24 h after birth (kg)	1.54±0.02^a^	1.38±0.02^b^	1.29±0.02^c^
Blood oxygen saturation (%)	88.7±0.9^a^	89.4±0.8^a^	89.2±0.9^a^
Colostrum consumption (g)	385.0±12.5^a^	330.8±10.9^b^	268.9±11.5^c^

SEM, standard error of the mean; TB, total number of piglets born per litter; BA, number of piglets born alive per litter; SB, number of stillborn piglets per litter; MF, number of mummified fetuses per litter.

a–c Different superscripts within each row differ significantly (p<0.05).

**Table 5:** Characteristics of sows and piglets that had normal parturition compared to those that required farrowing assistance (least square means±SEM)

Variables	Farrowing assistance		p-value

	No	Yes	
Sow characteristics			
Number of sows	247	105	
Parity number	4.4±0.1	4.4±0.1	0.767
Backfat thickness at 109 days of gestation (mm)	15.0±0.3	15.6±0.5	0.340
Farrowing duration (min)	241.5±15.7	308.7±23.9	0.019
Total number of piglets born per litter	15.1±0.1	15.2±0.2	0.791
Number of piglets born alive per litter	13.2±0.2	13.1±0.3	0.695
Number of stillborn piglets per litter	1.1±0.1	1.2±0.2	0.648
Number of mummified foetuses per litter	0.6±0.1	0.8±0.2	0.361
Colostrum yield (kg)	5.1±0.1	5.3±0.2	0.288
Litter birth weight (kg)	17.9±0.3	18.2±0.4	0.454
Variation of piglet birth weight (%CV)	21.5±0.4	20.8±0.7	0.404
Variation of colostrum consumption (%CV)	51.8±1.3	52.3±1.9	0.811
Piglet characteristics			
Number of piglets	5,089	465	
Birth interval (min)	13.8±0.9	20.6±1.8	<0.001
Cumulative birth interval (min)	109.4±4.2	169.0±8.7	<0.001
Blood oxygen saturation (%)	90.4±0.4	87.8±1.3	0.054
Piglet birth weight (kg)	1.29±0.01	1.30±0.03	0.673
Piglet body weight at 24 h after birth (kg)	1.40±0.01	1.41±0.03	0.791
Colostrum consumption (g)	354.2±5.6	302.2±15.7	<0.001

SEM, standard error of the mean.
